# Chemical and biochemical quality of organic and/ or mineral fertilization resources - A dataset from the Highlands of Madagascar

**DOI:** 10.1016/j.dib.2022.108458

**Published:** 2022-07-10

**Authors:** Manoa Raminoarison, Eric Blanchart, Tantely Razafimbelo, Laurent Thuriès, Jean Trap

**Affiliations:** a Laboratoire des Radio Isotopes, BP 3383, Route d'Andraisoro, Antananarivo 101, Madagascar; b Eco&Sols, Univ Montpellier, IRD, INRA, CIRAD, Montpellier SupAgro, Montpellier, France; c CIRAD, UPR Recyclage et Risque, F-97743 Saint-Denis, Réunion, France; d Recyclage et Risque, Univ Montpellier, CIRAD, Montpellier, France

**Keywords:** Nutrient contents, CO_2_ mineralization, Nitrogen mineralization, Ferralsols

## Abstract

A basic understanding of the fertilization resources (FR) characteristics is required to drive soil functions following the FR application, and to improve crop productivity. The datasets presented include the FR characteristics, i.e. their nutrient contents and biochemical quality, and their effects on soil in carbon (C) and nitrogen (N) mineralization. We selected nineteen FR from local farmers, from laboratory institution and from commercial producers. The soil used in experiment was sampled in Imerintsiatosika locality, located in the Central Highlands of Madagascar. Nutrient contents of FR were evaluated by measuring total carbon, nitrogen, phosphorus, potassium, calcium, magnesium and sulphur contents. Biochemical quality of the products was assayed by fractioning organic matter of organic resources in soluble compartments, hemicelluloses, celluloses and lignin equivalent. Laboratory incubations in microcosm experiments were conducted with the mixture of soil and fertilization resources to determine C and N mineralization rates. Carbon mineralization rate was measured using microgas chromatography, and nitrogen mineralization rates were analyzed by colorimetry on a continuous flow analyzer.

## Specifications Table

**Table:** 

Subject	Agricultural sciences
Specific subject area	Soil fertilization
Type of data	TablesFigures
How the data were acquired	Nineteen fertilization resources were collected in the beginning of the cultural season in November 2017 from local farmers, laboratory institution and commercial producers in the Highlands of Madagascar.The data on chemical composition of fertilization resources were acquired by using elemental analyzer, spectrophotometer, atomic absorption spectroscopy, glass electrode pH meter and fiber extraction.The data on carbon and nitrogen mineralization rates were acquired in laboratory by using a microcosm incubation. CO_2_ emitted was measured using a microgas chromatograph and mineral forms of N in the soil solution was measured using continuous flow analyzer.
Data format	RawAnalyzed
Description of data collection	Data collection was acquired in three steps:Step 1: Chemical and biochemical composition of organic and/ or mineral fertilization resources were determined using conventional methods. Analyses included moisture content, ash content, total carbon content, total nitrogen content, total sulphur content, total phosphorus content, total potassium content, total magnesium content, total calcium content, soluble content, hemicellulose-like content, cellulose-like content and lignin-like content.Step 2: Laboratory incubation were conducted using 150 mL hermetic glass jars for the determination of soil respiration following fertilization resources amendment. The incubation period lasted 150 days and CO_2_ microcosm air was measured 12 times after 1, 3, 7, 9, 14, 21, 34, 47, 56, 70, 95 and 150 days of incubation.Step 3: Plastic bottles of 50 mL were used and prepared to measure the kinetics of net nitrogen mineralization during 150 days. Mineral nitrogen extraction was carried out 5 times: 0, 15, 52, 95, 150 days after incubation.
Data source location	• Institution: Laboratory of Radio-Isotopes• City/Town/Region: Antananarivo• Country: Madagascar
Data accessibility	Data are included in this article and supplemented excel fileRepository name: FigshareData identification number: 10.6084/m9.figshare.19076969Direct link to the dataset: https://doi.org/10.6084/m9.figshare.19076969.v2

## Value of the Data

• Data on characteristics of fertilization resources from Madagascar's Highlands are scarce. The dataset presented herein provides the information necessary of chemical and biochemical quality of available fertilization resources and their effect on carbon and nitrogen mineralization when added in nutrient-poor tropical soils.
• This is of interest to soil scientists and agronomists working at the development of a decision support tool for the management of fertilization resources.
• These data can be used for designing innovative fertilization practices according to the quality of fertilization resources, such as development of combined fertilization to overcome soil nutrient constrains in smallholder systems.

## 1 Data Description

### 1.1 Chemical and biochemical composition

Description and origin of nineteen fertilization resources (FR) are presented in Table 1. The data includes nine types of products: three of plant ashes, five plant and animal composts, two household fermentable composts, three types of manure, one animal dejection without residues, two animal droppings, one crushed horn, one dolomite rock, and one phosphate fertilizer. Products were obtained from local farmers sited in Vakinankaratra and Itasy Region, from the Laboratory of Radio-Isotopes, Antananarivo University, Madagascar, and from commercial producers. This selection of fertilisation resources represents a large panel of products used by farmers and locally produced. Some products meet the NF U42-001 and NF U44-051 norms, referring respectively to ‘fertilizers’ and ‘organic amendments’ denominations. ‘Fertilizers’ contain at least 3 g 100g^−1^ bulk weight of a major nutrient (N, P_2_O_5_ or K_2_O), while ‘organic amendments’ denomination is for a product with an organic matter content is at least 20 g 100g^−1^ bulk weight and containing N, P_2_O_5_ or K_2_O less than 3 g 100 g^−1^ bulk weight.

**Table 1:** Description and origin of nineteen organic and/ or mineral fertilization resources available in the Highlands of Madagascar

Code	Class	Type	Norm	Description	Manufacturer
AshE	Binary PK fertilizers	Vegetable ashes	NF U42-001	Ash derived from combustion of *Eucalyptus* branches	Itasy farmer
AshH		Vegetable ashes		Rice husk ash obtained after burning out the husk in air	Itasy farmer
AshS		Vegetable ashes		Rice straw ash obtained after straw open-field burning	Vakinankaratra farmer
ComL	Organic amendments	Vegetable and animal composts	NF U44-051	Compost of mixing of: rice straw, *Hélianthus, Aristida sp.,*and farmyard manure (ManI product)	Laboratory of Radio Isotopes
VCL	Organic amendments	Vegetable and animal composts	NF U44-051	Vermicompost of mixing of: rice straw, *Hélianthus,Aristida sp.* and farmyard manure (ManI product), indigenous earthworms species: *Eudrilus eugeniae*	Laboratory of Radio Isotopes
VCT	Organic amendments	Vegetable and animal composts	NF U44-051	Vermicompost of mixing of: farmyard manure and disposal vegetables, imported earthworms species: *Eisenia foetida*	TATA association
VCV	Organic amendments	Vegetable and animal composts	NF U44-051	Vermicompost of mixing of: farmyard manure, soil, rice straw and legumes (mainly *Melia azedarac*), imported earthworms species: *Eisenia foetida* and *Eisenia andrei*	Vakinankaratra farmer
Tar		Vegetable and animal composts		Bacterial leaven fixed on a rich organic plant support, certified organic by Ecocert, commercialy known as Taroka Phosphaté	STOI Agri Company
ComM	Organic amendments	Household fermentable composts	NF U44-051	Fermentable composts from the dumpsite of Mahajanga municipal solid waste landfill under natural composting	Madacompost company
ComT		Household fermentable composts		Fermentable composts from the dumpsite of Andralanitra municipal solid waste landfill under natural composting	AKAMASOA Association
ManI	Organic amendments	Manures	NF U44-051	Fermentation of raw cattle dejections (excrements + urine) collected over vegetable residues mainly constitued of rice straw and prepared in outside rudimentary parc	Itasy farmer
ManV1	Organic amendments	Manures	NF U44-051	Fermentation of raw cattle dejections (excrements + urine) collected over vegetable residues mainly constitued of rice straw. The product is prepared and stored in closed place and collected in a specific recipient	Vakinankaratra farmer
ManV2	Organic amendments	Manures	NF U44-051	Fermentation of raw cattle dejections (excrements + urine) collected over vegetable residues mainly constitued of rice straw and prepared in outside rudimentary park	Vakinankaratra farmer
KM	Organic amendments	Animal dejections without residues	NF U44-051	Non fermentable mixing of soil and cattle dejections, collected in outdoor park and stored in the open air (kraal manure)	Vakinankaratra farmer
DroG	Entirely animal-based NP fertilizer	Bat guano droppings	NF U42-001	Dried droppings of bats. Product formed by accumulation and aging of those dropping birds, cave in the Southest Region	Guanomad company
DroP	Entirely animal-based NP fertilizer	Poultry droppings	NF U42-001	Product obtained by the accumulation and drying of poultry excrement for the production of commercial broilers	Itasy farmer
ZH	Nitrogen organic fertilizer	Crushed horn	NF U42-001	Product obtained by crushing and grinding zebu horns of different diameters: flour, semolina, shavings	Madacompost company
Dol	Fertilizer providing Ca, Mg, Na and/ or S	Dolomite	NF U42-001	Natural product essentially containing double carbonate of Ca and Mg, deposit in Ibity, Vakinankaratra Region	SEPCM Companies
Hyp	Phosphorus fertilizer	Rock phosphate + Volcanic black soil		Organo-mineral product obtained from mixing micronised apatitic rock and volcanic black soil, known commercially as Hyperfos	Prochimad Company

**Fig. 1:**
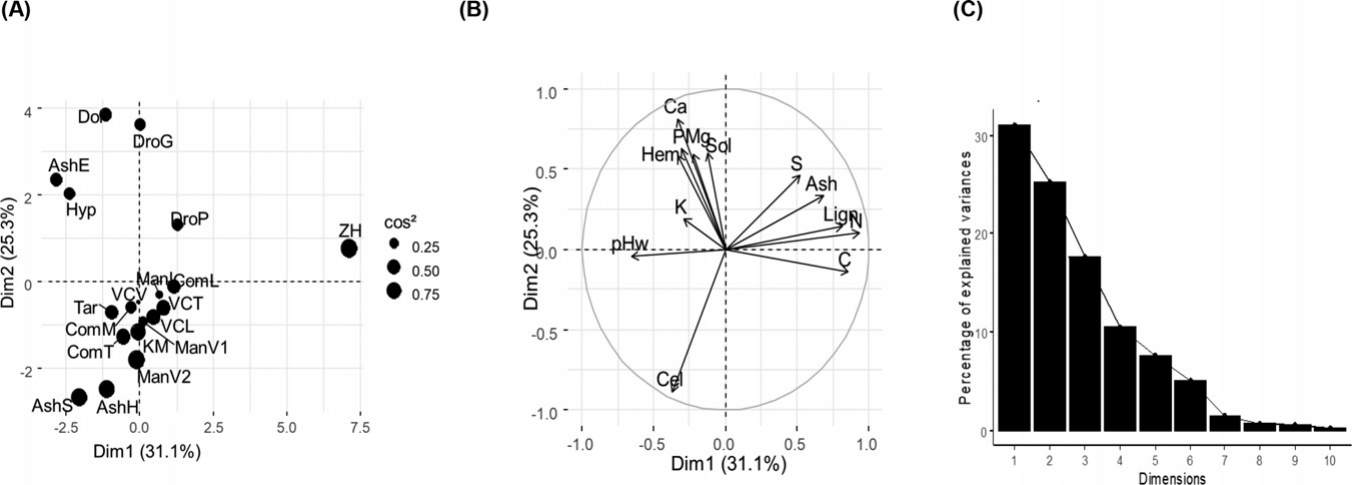
Principal Component Analysis performed on chemical and biochemical compositions of: (A) individual factorial plan (axes 1 and 2), (B) Correlation circle, and (C) Eigenvalue diagram. Ash: Ash content; C: Total organic carbon content; N: Total organic nitrogen content; P: Total phosphorus content; K: Total potassium content; Ca: Total calcium content; Mg: Total magnesium content; S: Total sulfur content; pHw: pH in water; Sol: Soluble content; Hem: Hemicelullose-like content; Cel: Celullose-like content; Lign: Lignin-like content **AshE**: Eucalyptus ash, **AshH**: rice ash husk, **AshS**: rice ash straw, **ComL**: Plant and animal composts by Laboratory of Radio Isotopes, **VCL**: Plant and animal vermicomposts by Laboratory of Radio Isotopes, **VCT**: Vermicompost of mixing of: farmyard manure and various plant residues by TATA Association, **VCV**: Plant and animal vermicomposts from Vakinankaratra farmer, **Tar**: Plant and animal composts by STOI Agri (Taroka), **ComM** and **ComT**: Fermentable composts from, respectively, the dumpsite of Mahajanga and Andranalanitra municipal solid waste landfill under natural composting. **ManI**: Manures from Itasy farmer, **ManV1** and **ManV2**: Manures from Vakinankaratra farmers, **KM**: Animal dejections without residues (kraal manure), **DroG**: Bat guano droppings, **DroP**: Poultry droppings, **ZH**: crushed zebu horn **Dol**: dolomite, **Hyp**: mixture phosphate rock and volcanic black soil.

To visualize the global chemical and biochemical variability of fertilization resources, a Principal Component Analysis (PCA) was carried out on chemical and biochemical composition (Fig. 1). The PCA exhibited a strong variability in chemical and biochemical composition among the 19 FR (Fig. 1). The first dimension (Dim1: 31.1% of relative inertia) opposed the crushed zebu horn (positive scores) to the other FR. This product was characterized by high values in total organic carbon, total organic nitrogen and lignin-like compounds which corresponds to keratin. The negative scores of this first dimension were characterized by high pH_w_ values. The second dimension (Dim2: 25.3 %) was mainly explained by total calcium and total phosphorus contents. It was associated to bat guano droppings. Four products including manure from Vakinankaratra (ManV2), kraal manure, husk ash and straw ashes had negative scores and were characterized by high content of cellulose-like fraction. On the third axis, dolomite showed high content of total magnesium, eucalyptus ash and manure from Vakinankaratra (ManV1) contributed to the last component due to a high value of total potassium content. PCA resulted in four principal components which explained 84 % of total inertia.

### 1.2 Potential CO_2_ mineralization

**Fig. 2:**
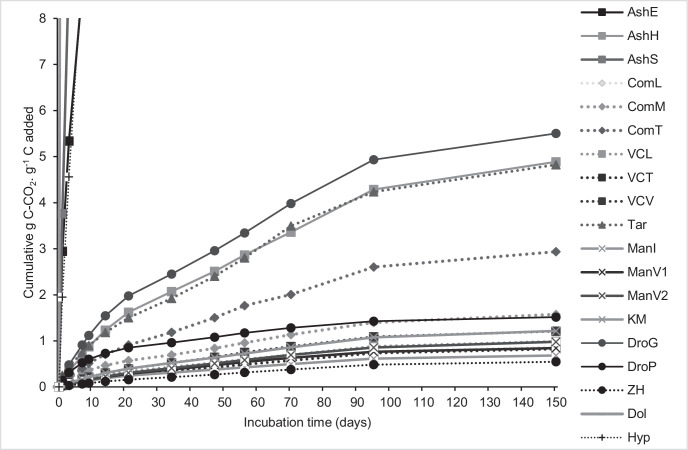
Cumulative C mineralization rates of the 19 organic and mineral fertilization resources measured during 150 days of incubation (g C-CO_2_. g^−1^ C applied). **AshE**: Eucalyptus ash, **AshH**: rice ash husk, **AshS**: rice ash straw, **ComL**: Plant and animal composts by Laboratory of Radio Isotopes, **VCL**: Plant and animal vermicomposts by Laboratory of Radio Isotopes, **VCT**: Vermicompost of mixing of: farmyard manure and various plant residues by TATA Association, **VCV**: Plant and animal vermicomposts from Vakinankaratra farmer, **Tar**: Plant and animal composts by STOI Agri (Taroka), **ComM** and **ComT**: Fermentable composts from, respectively, the dumpsite of Mahajanga and Andranalanitra municipal solid waste landfill under natural composting. **ManI**: Manures from Itasy farmer, **ManV1** and **ManV2**: Manures from Vakinankaratra farmers, **KM**: Animal dejections without residues (kraal manure), **DroG**: Bat guano droppings, **DroP**: Poultry droppings, **ZH**: crushed zebu horn **Dol**: dolomite, **Hyp**: mixture phosphate rock and volcanic black soil.

The amount of cumulated C mineralization along the incubation period ranged from 0.55 g C-CO_2_.g^−1^ C applied, corresponding to zebu horn, to 149 g C-CO_2_.g^−1^ C applied, corresponding to rice straw ash (Fig. 2). For seven products, recorded with the zebu horn, manure from Itasy farmer, vermicompost by TATA Association, the two manures from Vakinankaratra farmer (ManV1 and ManV2), compost and vermicompost produced by the Laboratory of Radio Isotopes, the cumulative C-CO_2_ evolved over the 150-day experiment did not reach 1 g C-CO_2_.g^−1^ C applied (averaged 0.55 to 0.99 g C-CO_2_.g^−1^ C applied). Inversely, eucalyptus ash, Hyperfos^TM^, straw ash led to extremely higher C mineralization rates, between 32.14 and 149 g C-CO_2_.g^−1^ C applied at the end of incubation. Values exceeding 1 g C-CO_2_.g^−1^ C applied. The remaining products exhibited C mineralization rates between 1.21 to 5.50 g C-CO_2_.g^−1^ C applied and correspond to vermicompost from Vakinankaratra farmer, kraal manure, poultry droppings, the two household fermentable composts, Taroka compost, husk ash, and bat guano droppings.

### 1.3 Potential net N mineralization

The amount of mineralized N at day 0 was the highest with bat guano droppings (22.31 mg N.kg^−1^ soil) and poultry droppings (19.30 mg N.kg^−1^ soil), the lowest values were observed with the one manure from Vakinankaratra (ManV2; 8.57 mg N.kg^−1^ soil) and the kraal manure (9.23 mg N.kg^−1^ soil) (Table 2).

**Table 2:** Amount of nitrogen mineralized at t=0.

	Total mineralized N at t=0
	µg N.kg^−1^ soil
**AshE**	11210
**AshH**	10383
**AshS**	11656
**ComL**	11236
**VCL**	12325
**VCT**	18787
**VCV**	14401
**Tar**	11119
**ComM**	14974
**ComT**	11826
**ManI**	17841
**ManV1**	14444
**ManV2**	8567
**KM**	9233
**DroG**	22314
**DroP**	19300
**ZH**	12733
**Dol**	14889
**Hyp**	11159

**AshE**: Eucalyptus ash, **AshH**: rice ash husk, **AshS**: rice ash straw,

**ComL**: Plant and animal composts by Laboratory of Radio Isotopes, **VCL**: Plant and animal vermicomposts by Laboratory of Radio Isotopes, **VCT**: Vermicompost of mixing of: farmyard manure and various plant residues by TATA Association, **VCV**: Plant and animal vermicomposts from Vakinankaratra farmer, **Tar**: Plant and animal composts by STOI Agri (Taroka),

**ComM** and **ComT**: Fermentable composts from, respectively, the dumpsite of Mahajanga and Andranalanitra municipal solid waste landfill under natural composting.

**ManI**: Manures from Itasy farmer, **ManV1** and **ManV2**: Manures from Vakinankaratra farmers, **KM**: Animal dejections without residues (kraal manure),

**DroG**: Bat guano droppings, **DroP**: Poultry droppings,

**ZH**: crushed zebu horn

**Dol**: dolomite,

**Hyp**: mixture phosphate rock and volcanic black soil.

During the laboratory incubation, a net mineralization was observed for the first 15 days after the application of fertilization resources (Table 3): the highest values were obtained with bat guano droppings (2.13 mg N.kg^−1^ soil. day^−1^) and crushed zebu horn (2.18 mg N.kg^−1^ soil. day^−1^) while the lowest values were obtained with poultry droppings (0.27 mg N.kg^−1^ soil. day^−1^). The mineralization rate after 52 and 95 days of incubation varied then largely. Again, crushed zebu horn led to greater N mineralized N (1.18 mg N.kg^−1^ soil. day^−1^) whereas bat guano droppings and one manure from Vakinankaratra (ManV1) led to N immobilisation (-0.57 and -0.25 mg N.kg^−1^ soil. day^−1^, respectively), at 52 days. At 95 days after incubation, net N immobilisation for nine fertilization resources were measured ranging from -0.40 to -0.01 mg N.kg^−1^ soil. day^−1^. The remaining products showed net N mineralization with values ranging from 0.02 (poultry droppings) to 0.67 mg N.kg^−1^ soil. day^−1^ (bat guano droppings). At the end of the incubation (150 days after incubation), the highest mineralized N were observed with the crushed zebu horn (0.49 mg N.kg^−1^ soil. day^−1^) and compost from the dumpsite of Andralanitra (0.47 mg N.kg^−1^ soil. day^−1^), whereas three fertilization resources induced net N immobilization (Dolomite, bat guano droppings and compost by Laboratory of Radio Isotopes) with respectively, -0.40, -0.38 and -0.08 mg N.kg^−1^ soil. day^−1^.

**Table 3:** Nitrogen mineralization rate in soils amended with nineteen fertilization resources after 15-, 52-, 95 and 150-days of incubation.

		15	52	95	150
		
		µg N.kg^−1^.day^−1^			
**AshE**	mean	1630.45	745.46	124.17	223.56
	SD	12.65	39.56	34.17	31.56
**AshH**	mean	1803.45	577.64	-30.76	94.89
	SD	11.98	13.61	23.32	13.24
**AshS**	mean	1674.81	575.45	-51.98	162.24
	SD	62.44	40.46	20.55	15.92
**ComL**	mean	1918.18	505.82	44.04	-80.83
	SD	6.86	18.60	19.70	92.14
**VCL**	mean	1803.38	469.18	-59.82	237.47
	SD	58.82	28.59	50.93	47.82
**VCT**	mean	1954.84	476.03	80.10	104.91
	SD	57.09	24.97	32.64	5.65
**VCV**	mean	1201.47	747.07	58.63	101.32
	SD	91.02	49.04	19.58	15.99
**Tar**	mean	1793.83	423.51	66.92	106.89
	SD	45.62	47.02	30.79	12.71
**ComM**	mean	1766.74	539.87	-124.45	226.94
	SD	35.32	18.12	5.06	17.29
**ComT**	mean	1781.80	535.67	-403.07	465.45
	SD	77.02	72.30	45.61	40.17
**ManI**	mean	1931.86	506.65	-8.39	157.21
	SD	55.08	55.49	50.46	38.68
**ManV1**	mean	1878.51	-245.62	526.94	129.79
	SD	34.83	221.73	149.44	13.31
**ManV2**	mean	1970.58	143.13	173.00	135.18
	SD	55.68	30.73	1.34	37.61
**KM**	mean	1888.46	487.11	-40.99	167.63
	SD	16.77	39.80	14.10	9.12
**DroG**	mean	2171.45	-570.89	668.69	-378.32
	SD	32.75	112.59	72.25	41.70
**DroP**	mean	273.03	643.14	18.05	335.51
	SD	26.75	9.14	8.04	29.92
**ZH**	mean	2133.68	1175.14	-87.73	488.08
	SD	20.39	31.31	75.40	60.90
**Dol**	mean	1917.17	565.37	140.10	-397.10
	SD	7.76	38.48	33.51	170.65
**Pho**	mean	1731.23	577.40	-109.20	145.32
	SD	7.21	3.54	40.37	28.01

**SD:** standard deviation

**AshE**: Eucalyptus ash, **AshH**: rice ash husk, **AshS**: rice ash straw,

**ComL**: Plant and animal composts by Laboratory of Radio Isotopes, **VCL**: Plant and animal vermicomposts by Laboratory of Radio Isotopes, **VCT**: Vermicompost of mixing of: farmyard manure and various plant residues by TATA Association, **VCV**: Plant and animal vermicomposts from Vakinankaratra farmer, **Tar**: Plant and animal composts by STOI Agri (Taroka),

**ComM** and **ComT**: Fermentable composts from, respectively, the dumpsite of Mahajanga and Andranalanitra municipal solid waste landfill under natural composting.

**ManI**: Manures from Itasy farmer, **ManV1** and **ManV2**: Manures from Vakinankaratra farmers, **KM**: Animal dejections without residues (kraal manure),

**DroG**: Bat guano droppings, **DroP**: Poultry droppings,

**ZH**: crushed zebu horn

**Dol**: dolomite,

**Hyp**: mixture phosphate rock and volcanic black soil.

## 2 Experimental Design, Materials and Methods

A total of nineteen fertilization resources (FR) was obtained from local farmers sited in Vakinankaratra and Itasy Region, from the Laboratory of Radio-Isotopes, Antananarivo University, Madagascar, and from commercial producers. For non-commercialized products, a total of ∼ 20 kg in fresh weight were randomly sampled in the storage place. For commercialized products, we purchased a bag of fertilizer at the sales points. After collection, FR were air-dried during seven days, manually grinded, sieved at 0.2 mm and stored in a cool and dry place. All fertilizers were subjected to three types of experimental analyses, operated to characterize each FR. The first analysis concerned their chemical and biochemical analytical compositions. The second was the analysis of C mineralization kinetics in controlled conditions. The last corresponded to the measurement of net N mineralization.

Each sample of air-dried FR was homogenized by hand during 10 min and separated into aliquots for various chemical and biochemical analyses. The moisture content was determined by drying in an oven at 105°C. Ash content was measured by determining loss on ignition at 550°C for 4 h [1]. Other aliquots were dried at 40°C and the total C, N and S content was evaluated by dry combustion at Flash 2000 CHN Analyzer (Flash 2000 Series. CHNS / O 122 Thermo Scientific Analyzers, IRCOF, France). Total P was extracted by calcination in the oven at 550°C for 5 h and digested with hydrochloric acid (0.5 g samples) 2%, and assayed by colorimetry with molybdenum blue by spectrophotometer [2]. A subsample of the same digest was used for the assay of total K, Ca and Mg by atomic absorption spectroscopy (iCE 3000 Series AA Spectrometer. Belgium) [3]. The pH of the products was measured using a glass electrode pH meter (pH 211 Microprocessor pH Meter. Romania) in the ratio 1:5 (FR:water). Biochemical quality of fertilization resources was obtained using the Van Soest biochemical fractionation [4] as described in the standardized method (FD U44-162). In this method, successive extractions with neutral detergent (NDF), acid detergent (ADF) and lignin acid detergent (ADL) are used to discretise the non-soluble organic matter into 4 fractions: water soluble organic matter and soluble in neutral detergent (SOL), hemicellulose-like organic matter (HEM), cellulose-like organic matter (CEL) and lignin-like organic matter (LIG).

A Principal Component Analysis (PCA) was performed with all fertilization resources using R software v. 3.5.1 (R Core Team, 2018) with factoextra::PCA function. The PCA was conducted using thirteen chemical and biochemical variables, i.e. ash content, total organic carbon content, total organic nitrogen content, total phosphorus content, total potassium content, total calcium content, total magnesium content, total sulfur content, pH in water, soluble content, hemicelullose-like content, celullose-like content and lignin-like content of the 19 FR.

The kinetic of C and N mineralization of FR were conducted on the same experimental soil under controlled conditions (FD U44-163). The soil was sampled from the upper layer (0-20 cm) of one site left under natural savannah dominated by bozaka, located in the Eastern part of the Itasy region, near the locality of Imerintsiatosika (19°05’40’’S; 47°25’65’’E; 1480 m above sea level). Soil is classified in the FAO Classification as Ferralsols [5]. The soil samples were air-dried, passed through a 2-mm sieve, before analysis of selected properties and for the incubation study. Soil had acidic pH (<5), low Olsen P contents ca. 3.8 mg.kg^−1^, low CEC (< 2 cmol^+^ kg^−1^) and very low exchangeable cations (K^+^, Ca^2+^, Mg^2+^). Low total N contents was also observed (<0.25%). Soil organic carbon content was in average 29.2 g kg^−1^. Its texture was dominated by fine fractions (clay + fine silt: 71.3%).

Laboratory incubations were conducted using 150 mL hermetic glass jars for the determination of soil respiration following FR amendment. A total of 57 microcosms were prepared corresponding to the study of 19 FR with three replicates. Twenty grams of dry soil were weighed and placed in the microcosms. The soil was then wetted with distilled water to bring the moisture to 80% of its water holding capacity (WHC) and pre-incubated for seven days at 27°C in darkness to restore the microbial activity [6]. Afterwards, an equivalent of 0.004 g of FR per g of dry weight soil was mixed with the soil, giving a quantity of 80 mg of FR per microcosms. The humidity of soil-FR mixtures was maintained at 80% WHC during the experiment. Each was then sealed hermetically. The incubation period lasted 150 days and CO_2_ microcosm air was measured 12 times after 1, 3, 7, 9, 14, 21, 34, 47, 56, 70, 95 and 150 days. CO_2_ was determined using a Varian CP4900 microgas chromatograph (Varian Chromatography Group, Walnut Creek, CA USA). After each CO_2_ measurement, jars were opened, aerated and closed before incubation. The rate of CO_2_ emission was expressed as g of C per gram added total organic carbon (TOC). The cumulative C-CO_2_ emitted over the 150-day incubation was then calculated.

Plastic bottles of 50 mL were used and prepared to measure the kinetics of net N mineralization during 150 days. The same experimental procedure and conditions as CO_2_ measurement were used. except for the pre-incubation step which was excluded. The dry soil weight was reduced to 10 g per bottles with the same ratio dry FR weight: dry soil weight of 0.4%. Mineral N content was carried out 5 times: 0, 15, 52, 95, 150 days after incubation. Three replications of each treatment (19 additions of FR) for each sampling date (except extraction at T0) were used so that destructive sampling could be carried out for a total of 247 jars. Soil mineral N was extracted with 40 mL of 1M KCl after shaking the soil suspension for one hour and centrifugating it for 5 min at 3000 tours. min-1. The soil suspension was then filtrated using Whatman 40 filter paper. The concentrations of NH_4_^+^ and NO_3_^−^ in the filtrate were analyzed by colorimetry on a continuous flow analyzer (SKALAR) using the Berthelot method for the determination of N- NH_4_^+^ and the Griess and Ilossay method for the determination of N- NO_3_^−^ [7]. after reduction of nitrates to nitrites by passage over a column of cadmium. Net N mineralization was expressed in µg N.kg^−1^.day^−1^.Net N mineralization was calculated as the difference between the soil N concentration at each sampling period and the initial N concentrations. The following equations were used [8]:

$$\text{Potential}\;\text{net}\;\text{ammonification}\;=\;\left[\left({N-\text{NH}}_{4}^{+}\right)f\;-\;\left({N-\text{NH}}_{4}^{+}\right)i\right]/\text{Td}\;$$

$$\text{Potential}\;\text{net}\;\text{nitrification}\;=\;\left[\left({N-\text{NO}}_{3}^{-}\right)f\;-\;\left({N-\text{NO}}_{3}^{-}\right)i\right]/\text{Td}$$

Where i indicate the initial mineral N concentration, f the N concentration on a sampling period, and Td incubation time in days. A negative value indicates microbial net immobilization.

## CRediT Author Statement

**Manoa Raminoarison:** Conceptualization, Methodology, Writing, review & editing; **Eric Blanchart:** Conceptualization, Writing – review & editing; **Tantely Razafimbelo:** Conceptualization, Writing – review & editing; **Laurent Thuriès:** Methodology, Writing – review & editing; **Jean Trap:** Conceptualization, Methodology, Writing – review & editing.

## Ethic Statements

The authors declare that there are no ethical issues with the data presented and the methods used.

## Declaration of Competing Interest

The authors declare that they have no known competing financial interests or personal relationships that could have appeared to influence the work reported in this paper.

The authors declare the following financial interests/personal relationships which may be considered as potential competing interests:

## Data Availability

Chemical and biochemical quality of organic and/ or mineral fertilization resources - A dataset from the Highlands of Madagascar (Original data) (Dataverse).
